# STAM Prolongs Clear Cell Renal Cell Carcinoma Patients' Survival *via* Inhibiting Cell Growth and Invasion

**DOI:** 10.3389/fonc.2021.611081

**Published:** 2021-04-20

**Authors:** Tuo Deng, Zihao He, Xiaolu Duan, Di Gu, Chao Cai, Wenqi Wu, Yongda Liu, Guohua Zeng

**Affiliations:** Department of Urology and Guangdong Key Laboratory of Urology, The First Affiliated Hospital of Guangzhou Medical University, Guangzhou Medical University, Guangzhou, China

**Keywords:** stam, clear cell renal carcinoma, prognosis, cell growth & apoptosis, invasion

## Abstract

**Background:** Signal transducing adaptor molecule 1 (STAM1) was considered to mediate cell growth and be involved in multiple signaling pathways; however, no research on the role of STAM1 in any tumors has been published yet. Our study aimed to investigate the prognostic value of STAM1 for clear cell renal cell carcinoma (ccRCC) and its role in modulating cancer cell function.

**Methods:** Data from The Cancer Genome Atlas (TCGA) in December 2019 were used to examine the role of STAM1 in indicating ccRCC patients' survival. A purchased tissue microarray (TM) and fresh ccRCC renal tissues were used for further validation. Then, STAM1 was overexpressed in human ccRCC cell lines for *in vitro* assays. Finally, bioinformatics was performed for STAM1 protein–protein interaction (PPI) network construction and functional analyses.

**Results:** A total of 539 ccRCC and 72 control samples were included for the TCGA cohort, and 149 ccRCC and 29 control slices were included for the TM cohort. In the TCGA and TM cohorts, we found that STAM1 expression was lower in ccRCC compared with normal adjacent non-cancerous renal tissues (*P* < 0.0001 for both cohorts). STAM1 downregulation was also related to significantly shorter overall survival (OS) (*P* < 0.0001 for both cohorts). In the TCGA cohort, reduced STAM1 expression was also associated with aggressive features of the tumor. Under multivariate analyses, STAM1 was demonstrated to be an independent prognostic factor for ccRCC survival in both TCGA (HR = 0.52, 95% CI: 0.33–0.84, *P* = 0.007) and TM cohorts (HR = 0.12, 95% CI: 0.04–0.32, *P* < 0.001). Our *in vitro* experiments showed that STAM1 inhibited cell viability, invasion, and migration in ccRCC cell lines. In PPI network, 10 candidate genes categorized into five biological processes were found to be closely related to STAM1.

**Conclusion:** STAM1 is a promising prognostic biomarker for predicting ccRCC survival outcomes. Preliminary pathogenesis is demonstrated by our *in vitro* experiments. Further pathological mechanisms of STAM1 in modulating ccRCC require comprehensive laboratory and clinical studies.

## Introduction

Renal cell carcinoma (RCC) is the most common kind of kidney malignant tumor. Among more than 400,000 new cases of kidney cancer in each year, RCC is responsible for ~90% of them (1, 2). Clear cell RCC (ccRCC) is the most frequent histological form, accounting for 70–80% in patients bearing kidney cancer (3). The cure rate is relatively high when the tumor is early and localized; however, as an aggressive subtype, the 5-year survival of ccRCC drops to <12% when metastasis occurs (4). For advanced ccRCC, after the patients received total nephrectomy, about 30% cases suffer from tumor recurrence (5). Because all RCC subtypes lack sensitivity to chemotherapy and radiotherapy (6), novel treatments are required to be discovered necessarily by targeting specific molecular hallmarks, which play a pivotal role in the pathogenesis of the disease.

ccRCC is defined as “clear cell,” owing to lipid and glycogen accumulation in the cytoplasm. The inactivation of von Hippel Lindau tumor-suppressor gene, found in up to 90% of patients, is a representative biomarker (7), and its downstream signaling pathways are influenced accordingly, leading to tumor angiogenesis, cell migration, and proliferation (8). Although biotherapies have been studied widely, such as anti-angiogenetic agents and immune pathway blockers (9), resistance to these therapies is still often observed in ccRCC patients. Moreover, molecular prognostic factors related to the mechanisms of the disease need to be further investigated because the prognostic information from clinical and histological evidence is limited (10). Therefore, our main purpose aims to find a new biomarker, which is closely associated with the pathological process, to predict the prognosis of ccRCC.

Signal transducing adaptor molecule (STAM), originally named STAM1, was firstly purified in 1996 from a series of molecules which are rapidly tyrosine-phosphorylated after cytokine stimulation (11). Containing a Src homology 3 (SH3) domain and immunoreceptor tyrosine-based activation motif (ITAM), STAM1 was considered to mediate cell growth and be involved in multiple signaling pathways (12, 13). In 2000, a cDNA encoding STAM2 was also cloned, having similar structures and functions with STAM1 (14). In addition, both STAM1 and STAM2 were later proved to be important components of the endosomal sorting complex required for transport-0 (ESCRT-0) complex (15). Since tumor biogenesis and progression is a complicated process, including neo-angiogenesis, change of microenvironment, disturbance of endosomal transportation, and so on, STAM1 potentially participates in the pathogenesis of tumors. No research on the role of STAM1 in any tumors has been published yet. In our study, we focused on STAM1 and its relationship with ccRCC by RNA sequencing through online databases at first. Subsequently, we confirmed STAM1 function through experimental and clinical findings, further providing its value in predicting the prognosis of ccRCC.

## Materials and Methods

### TCGA ccRCC Cohort

RNA sequencing data and clinicopathological information based on publicly available ccRCC cohort were obtained from an independent online database in December 2019, namely, The Cancer Genome Atlas (TCGA, https://portal.gdc.cancer.gov/) (16). RNA-seq data retrieved from TCGA was normalized as fragments per kilobase per million value.

### Clinical Patient Cohort and Fresh Tissue Specimens

A tissue microarray (TM) was purchased from Shanghai Outdo Biotech Company (No. HKidE180Su02) for validation. ccRCC samples from 149 patients were obtained with their clinicopathological data, 29 of which were matched with normal adjacent non-cancerous renal tissues as controls. All included patients received surgeries from 2008 to 2010 and were followed up to August 2015. Additionally, six pairs of fresh ccRCC and controlled normal adjacent non-cancerous renal tissues from the First Affiliated Hospital of Guangzhou Medical University, China, were also achieved to verify STAM1 mRNA expression. Our study was approved by the Ethics Committee of the First Affiliated Hospital of Guangzhou Medical University, China. Fully informed consents were approved by the included patients.

### Immunohistochemical Staining

The primary antibody for immunohistochemical (IHC) staining was rabbit polyclonal anti-STAM1 antibody (1:100, #ab244470, Abcam), and the secondary antibody was horseradish peroxidase-conjugated goat anti-rabbit IgG (1:2,000, #ab205718, Abcam). The IHC staining process was performed on TM samples under the manufacturer's instructions. The STAM1 protein level in tissue sections was semi-quantified by two experienced pathologists using a scoring system based on staining intensity and extent. The staining intensity scores were defined as 0 (no staining), 1 (weak positive), 2 (moderate positive), and 3 (strong positive). The immunoreactive scores were evaluated according to the percentage of positive cells: 0 (negative), 1 (1–25%), 2 (26–50%), 3 (51–75%), and 4 (76–100%) points by using the IHC Toolbox plugin in ImageJ software (17–19). The IHC scores were integers ranging from 0 to 12, which represented STAM1 protein level in tissue slices and were utilized for subsequent analyses.

### Cell Culture and Transfection

Human RCC cell lines (786-O and A498) were purchased from the American Type Culture Collection and cultured in Dulbecco's modified Eagle's medium (DMEM; Invitrogen) supplemented with 10% fetal bovine serum (FBS) at 37°C in a humidified incubator containing 5% CO_2_. Human embryonic kidney (HEK) 293 cells were maintained in DMEM with 10% FBS, 1% penicillin/streptomycin, and 10 mmol/L HEPES buffer.

To achieve STAM1 overexpression in cell lines, full-length STAM1 was cloned into a modified pcDNA3.1 vector in-frame with hemagglutinin (HA). For transient transfections, cells were seeded in 60-mm dishes and transfected at 70% confluence. The transfections were conducted with plasmids using Lipofectamine LTX according to the manufacturer's instructions (Invitrogen).

### Quantitative Reverse-Transcription Polymerase Chain Reaction

Quantitative reverse-transcription polymerase chain reaction (qRT-PCR) was performed on six pairs of fresh samples from our hospital and in cell experiments. After isolating RNA from tissues and cells using TRIzol Reagent (Invitrogen), RNA was reversely transcribed to complementary DNA for amplification. The STAM1 primer was designed as follows: forward: 5′-AATCCCTTCGATCAGGATGTTGA-3′, reverse: 5′-CGAGACTGACCAACTTTATCACA-3′. GAPDH served as set as internal reference (forward: 5′-AGAAGGCTGGGGTCATTTG-3′, reverse: 5′-GCAGGAGGCATTGCTGATGAT-3′). The STAM1 mRNA level was normalized and quantified by BioRad CFX Manager software.

### Western Blot Analysis

STAM1 protein expression in cell lines was detected by western blot analysis according to the protocol. The antibodies in the present study were anti-STAM1 (polyclonal rabbit, cat. no. ab244470, Abcam, USA) and anti-GAPDH (polyclonal rabbit, cat. no. 10494-1-AP, Proteintech, USA).

### Cell Viability Assay

A total of 5 × 10^3^ cells were seeded in 96-well plates and cultured for 24, 48, and 72 h, respectively. The cells were then incubated with 10 μl of CCK-8 solution (#C0039; Beyotime, China) for 2 h at 37°C. The absorbance was measured at a wavelength of 450 nm with a spectrophotometer. All results were expressed as means ± SD of three independent experiments.

### Cell Apoptosis Assay

Apoptosis was measured through Annexin V-APC/7-AAD double staining. For flow cytometry, 1 × 10^6^ 786-O or A498 cells were washed with cold phosphate-buffered saline (PBS), centrifuged, and resuspended with 1 × binding buffer. Each 100-μl cell suspension was stained with 5 μl Annexin V-APC and 10 μl 7-AAD (cat. no. 4224750, MultiSciences, Hangzhou, China) at room temperature for 15 min, respectively. Then, 385 μl of cold 1 × binding buffer was added to the cells, and the cells were analyzed by flow cytometry using BD FACSCalibur flow cytometer (Becton & Dickinson Company, Franklin Lakes, NJ, USA). Each experiment was performed three times. Cells with Annexin V-APC/7-AAD staining were considered in early-stage apoptosis, while cells with Annexin V-APC + /7-AAD + or Annexin V-APC − /7-AAD + staining were identified in late-stage apoptosis or necrosis.

### Transwell Assay

In this assay, 5 × 10^4^ transfected cells were placed in the upper chamber of Transwell plates pre-coated with Matrigel and cultured in serum-free medium, while the lower chamber contained a culture medium with 20% FBS acting as a chemoattractant. After 24-h incubation at 37°C in 5% CO_2_, cells on the undersurface of the filter were fixed with methanol, stained with 0.1% crystal violet, and counted under a microscope. All experiments were conducted at least three times.

### Wound-Healing Assay

Wound-healing assay was conducted to assess cell migration ability. Transfected 786-O and A498 cells with control groups were seeded in six-well-plates (1 × 10^6^ cells/well) with three replicates in each condition and cultured to sub-confluence in the complete medium. After 24-h starvation in serum-free medium, a linear artificial wound was scraped in the confluent cell monolayer with a standard P-200 pipette tip. Cells detached from the bottom of the wells were gently washed by warm PBS and aspirated. At 0, 4, and 8 h, the width of the scratch gap was observed under an inverted microscope and photographed. Quantification was performed by measuring the number of pixels in the wound area using ImageJ software. Three replicates were set in each condition.

### Protein–Protein Interaction Network Construction and Functional Analyses

The construction of protein–protein interaction (PPI) network was realized *via* the Search Tool for the Retrieval of Interacting Genes (STRING; https://string-db.org/) online database, which contributed to a better understanding of functional interactions among gene products (20, 21). As previously reported, interactions reaching “medium confidence” (or a combined score of >0.4) were regarded as significant in the PPI network (22). Therefore, we adopted a combined score of 0.4 as the criteria to screen the candidates for PPI network construction. Particularly, genes encoding proteins with top 10 combined scores were selected and incorporated into the PPI network.

Further analyses and interpretations concerning STAM1 were implemented Gene Ontology (GO) functional annotation and Kyoto Encyclopedia of Genes and Genomes (KEGG) pathway enrichment through online Database for Annotation Visualization and Integrated Discovery (https://david.ncifcrf.gov/) (23). GO terms and pathways with *P* < 0.05 and gene count ≥2 were considered as significant. Outcome visualization of gene expression and GO/KEGG enrichment analyses were achieved using the “pheatmap” and “ggplot2” packages in R software.

### Statistical Analysis

Mann–Whitney *U*-test was used to compare STAM1 expression in different groups. In the survival analyses, optimal cutoff values of STAM1 expression were identified using X-tile software as described previously (24). Kaplan–Meier survival analysis along with log-rank test was applied to evaluate the impact of STAM1 expression on ccRCC patients' overall survival (OS), defined as the time span from the onset of follow-up to death of any cause or the end of follow-up. Samples lacking necessary clinicopathological data were excluded. To analyze the association between STAM1 expression and clinicopathological features, chi-square test was adopted. Univariate and multivariate Cox regression analyses were employed to identify independent prognostic factors for ccRCC OS from variables including STAM1 expression, age, gender, TNM stage, grade, and American Joint Committee on Cancer (AJCC) stage. Stepwise regression was used in multivariate analyses. All statistical analyses were conducted using Prism GraphPad 7 software and R software (version 3.6.0). Student's *t*-test was applied in analyzing our fresh tissue samples and cell experiments. A two-sided *P* < 0.05 was regarded as statistically significant.

## Results

### Characteristics of ccRCC Cohorts

In the present study, a ccRCC cohort from the TCGA public database was included for bioinformatic analyses, and the TM cohort was added for validation. A total of 539 ccRCC and 72 control samples were included for the TCGA cohort, and 149 ccRCC and 29 control slices were included for the TM cohort. The clinicopathological characteristics of ccRCC patients with survival data in the TCGA and TM cohorts are summarized in Table 1. The data of 530 ccRCC patients in the TCGA cohort and 149 ccRCC patients in the TM cohort were used for survival analyses. All 149 patients in the TM cohort were diagnosed as primary unilateral ccRCC (mean age, 56.91 ± 11.55 years), and follow-up time ranged from 4 to 90 (68.16 ± 21.36) months.

**Table 1:** Characteristics of ccRCC patients from The Cancer Genome Atlas (TCGA) and tissue microarray cohorts.

		**TCGA cohort**					**Tissue microarray cohort**				
			**STAM1 expression**		**χ^**2**^**	***P*-value**		**STAM1 expression**		**χ^**2**^**	***P*-value**
			**High**	**Low**				**High**	**Low**		
Age (mean ± SD, years)		60.56 ± 12.14	–	–	–	–	56.91 ± 11.55	–	–	–	–
Gender	Male	344	265	79	2.42	0.12	106	72	34	0.05	0.83
	Female	186	154	32			43	30	13		
pT stage	T1–T2	340	283	57	10.00	0.0016	138	95	43	0.00	0.98
	T3–T4	190	136	54			11	7	4		
pN stage	N0	239	190	49	2.56	0.11	146	100	46	–	>0.99
	N1	16	10	6			3	2	1		
pM stage	M0	420	339	81	3.19	0.07	149	102	47	–	–
	M1	78	56	22			0	0	0		
Grade	G1–G2	241	206	35	10.96	0.0009	84	72	12	0.50	0.48
	G3–G4	281	207	74			36	29	7		
AJCC stage	I–II	322	273	49	16.03	<0.0001	137	94	43	0.03	0.85
	III–IV	205	144	61			12	8	4		
Total patients	530	419	111	–	–	149	102	47	–	–

*ccRCC, clear cell renal cell carcinoma; SD, standard deviation*.

The ccRCC patients in each cohort were categorized into STAM1 high- or low-expression group according to the optimal cutoff values of STAM1 expression in total OS analyses (cutoff values: 4.10 in the TCGA cohort and 1.00 in the TM cohort; Supplementary Table 1). In addition, the clinicopathological information of six ccRCC patients from our hospital is listed in Supplementary Table 2.

### Low STAM1 Level Correlated With Poor Prognosis in TCGA Cohort

**Figure 1:**
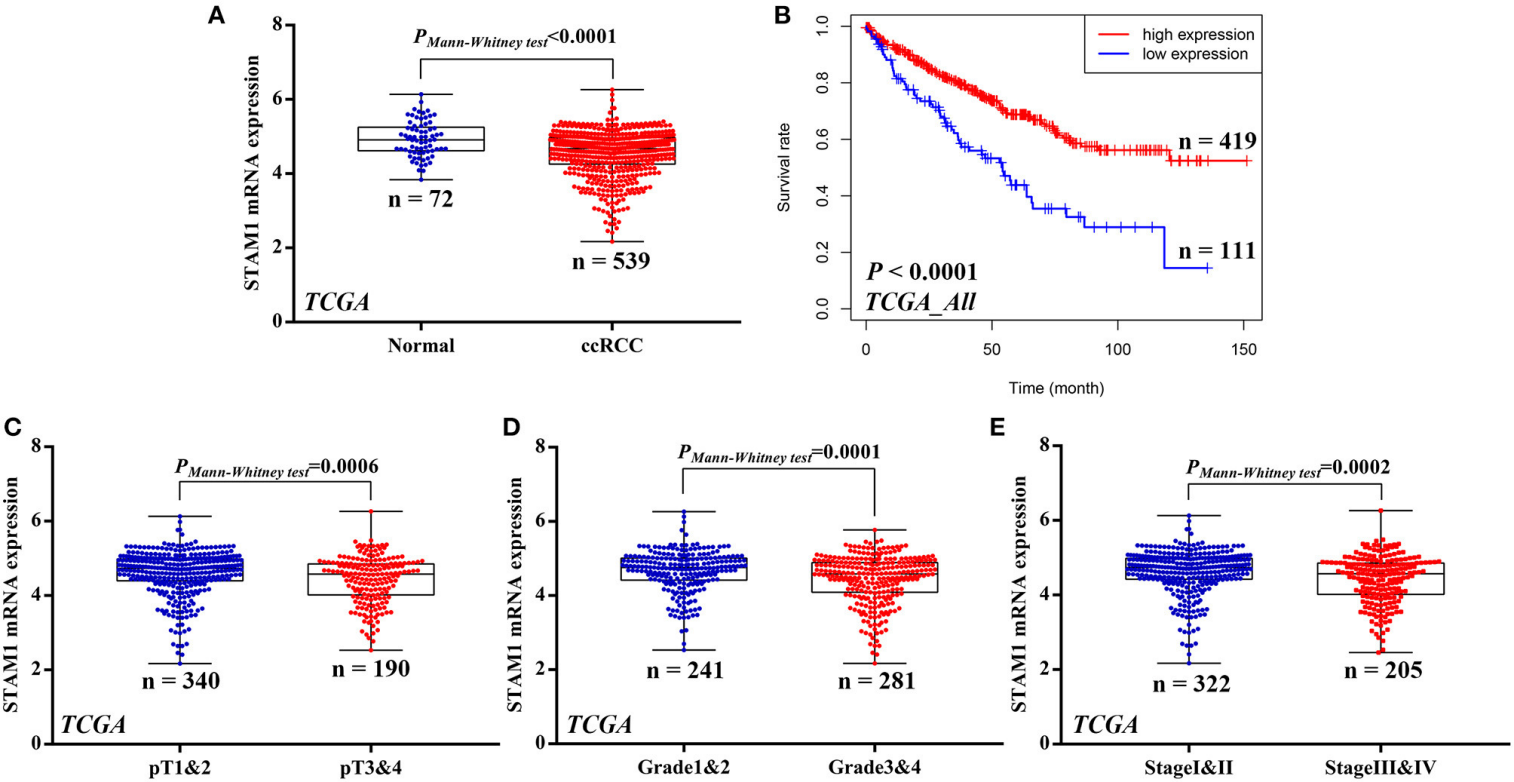
mRNA expression of STAM1 and its association with survival in clear cell renal cell carcinoma (ccRCC) based on The Cancer Genome Atlas data mining. **(A)** Comparison of STAM1 mRNA level between ccRCC tissues and cancer-adjacent normal renal tissues. **(B)** Kaplan–Meier analysis of overall survival in patients with low and high STAM1 level. Comparisons of STAM1 mRNA levels in ccRCC patients with low and high pT stages **(C)**, tumor grades **(D)**, and American Joint Committee on Cancer stages **(E)**.

To investigate whether STAM1 was differently expressed between ccRCC and controlled normal adjacent non-cancerous renal tissues, the accessible expression data of 539 ccRCC samples and 72 controls from TCGA database was initially analyzed. The results demonstrate that the STAM1 mRNA level was significantly lower in ccRCC tissues compared with controls (*P* < 0.0001) (Figure 1A). Next, survival analyses from TCGA cohort incorporating 530 cases revealed that lower STAM1 expression was related to significantly shorter OS (*P* < 0.0001) (Figure 1B).

Subsequently, associations of STAM1 mRNA level and tumor T stage, grade, and AJCC stage were evaluated based on the TCGA cohort. By comparing STAM1 expression between different ccRCC T stages (pT1 and 2 *vs*. pT3 and 4) (*P* = 0.0006, Figure 1C), tumor grade (grade 1 and 2 *vs*. grade 3 and 4) (*P* = 0.0001, Figure 1D), and AJCC stages (stage I and II *vs*. stage III and IV) (*P* = 0.0002, Figure 1E), the outcomes indicated that a low STAM1 level was significantly correlated with the aggressive features of ccRCC.

**Figure 2:**
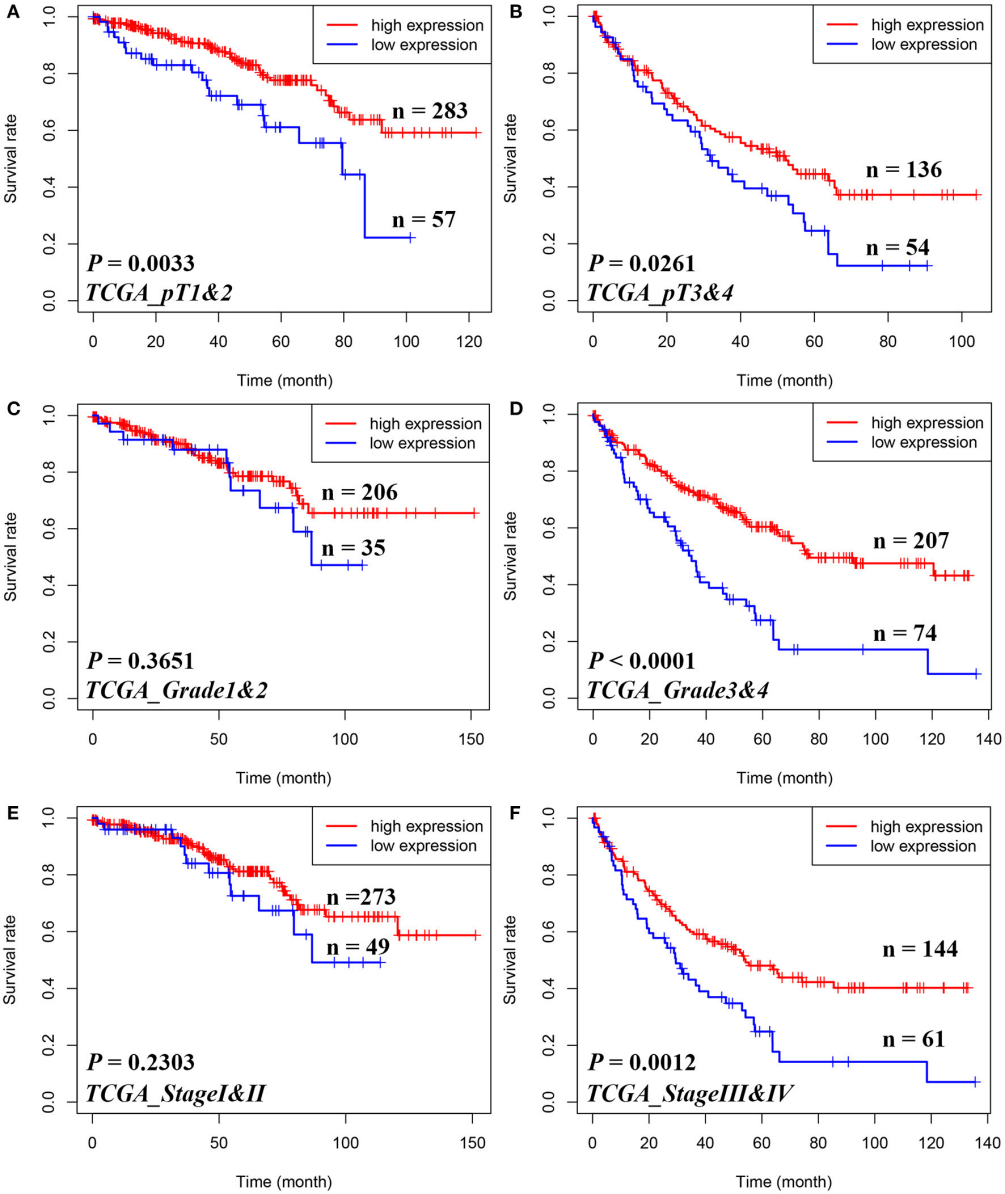
Subgroup analyses to assess the prognostic value of STAM1 based on pT stages, tumor grades, and American Joint Committee on Cancer (AJCC) stages in clear cell renal cell carcinoma patients from The Cancer Genome Atlas. Kaplan–Meier analyses of overall survival in patients with pT1 and 2 **(A)**, pT3 and 4 **(B)**, grade 1 and 2 **(C)**, grade 3 and 4 **(D)**, AJCC stage I and II **(E)**, and AJCC stage III and IV **(F)**.

Furthermore, in Figure 2, Kaplan–Meier analyses of OS demonstrated that STAM1 downregulation is an ideal biomarker in predicting worse survival in progressed ccRCC patients compared with early-stage cancer. Significant outcomes included pT1 and 2 (*P* = 0.0033), pT3 and 4 (*P* = 0.0261), grade 3 and 4 (*P* < 0.0001), and AJCC stage III and IV (*P* = 0.0012).

### Verifications by TM Cohort and Fresh Tissue Samples

**Figure 3:**
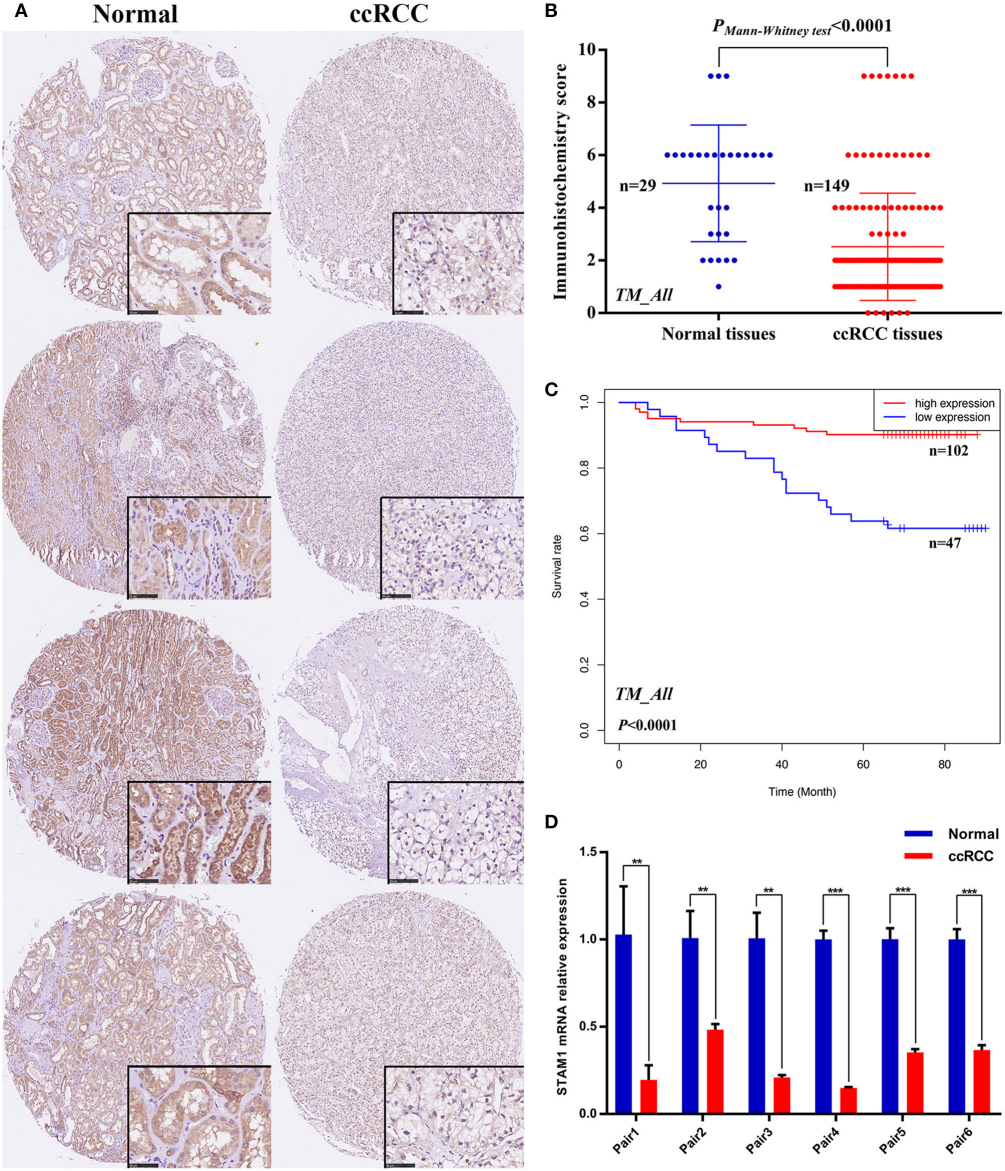
Protein and mRNA expression of STAM1 in tissue microarray (TM) cohort and fresh samples in our hospital; overall survival (OS) based on STAM1 levels in clear cell renal cell carcinoma (ccRCC) from TM cohort. **(A)** Immunohistochemical (IHC) staining sections for STAM1 of the representative four paired ccRCC tissues and cancer-adjacent normal renal tissues from TM cohort. **(B)** Comparison of IHC scores of STAM1 between ccRCC tissues and cancer-adjacent normal renal tissues in TM cohort. **(C)** Kaplan–Meier analysis of OS in patients with low and high STAM1 level in TM cohort according to IHC scores. **(D)** Comparison of STAM1 mRNA level in six paired fresh ccRCC tissues and cancer-adjacent normal renal tissues from our hospital through polymerase chain reaction. **p < 0.01, and *** *p* < 0.001.

IHC staining on tissues from the TM cohort and qRT-PCR on six paired fresh tissue samples from our hospital were performed to confirm the above-mentioned bioinformatic findings. As shown in Figure 3A, four pairs of ccRCC sections and controls in the TM cohort were selected as representative images, illustrating the subcellular localization of STAM1 protein that included both the nucleus and the cytoplasm, with remarkably more abundant staining in the latter. Based on IHC scores of 149 ccRCC and 29 control slices, significant downregulation of STAM1 in ccRCC tissues was corroborated at the protein level (*P* < 0.0001, Figure 3B). Survival analysis further verified that the low expression of STAM1 was related to significantly poor OS (*P* < 0.0001, Figure 3C). Finally, qRT-PCR results indicated significantly reduced STAM1 expression at the mRNA level in ccRCC tissues compared with controls (*P* < 0.001, Figure 3D).

**Figure 4:**
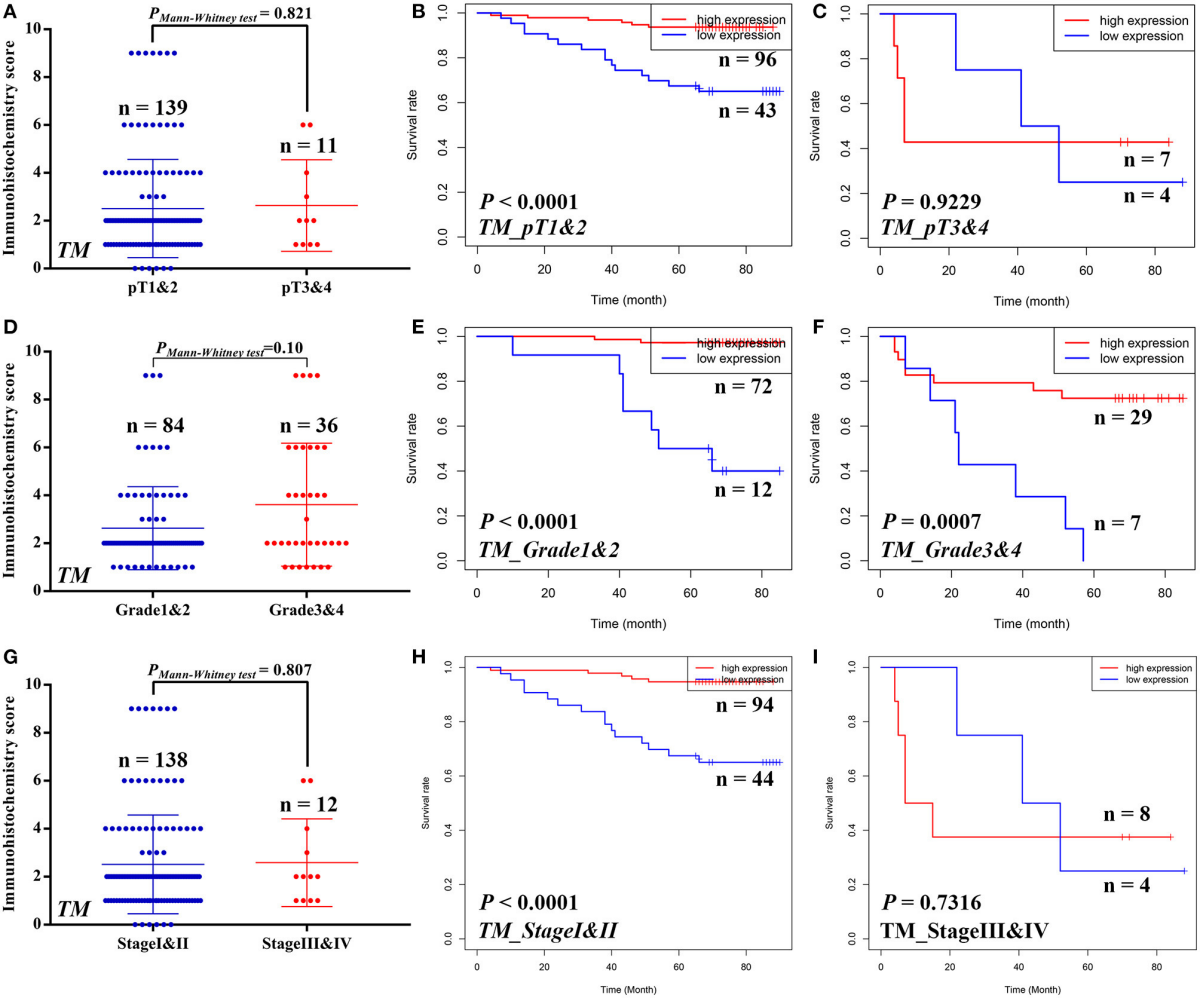
Subgroup analyses to compare the protein expression and assess the prognostic value of STAM1 based on pT stages, tumor grades, and American Joint Committee on Cancer (AJCC) stages in clear cell renal cell carcinoma (ccRCC) patients from tissue microarray cohort. Comparison of STAM1 protein level in ccRCC patients with low and high pT stages **(A)** and Kaplan–Meier analyses of overall survival (OS) in patients with pT1 and 2 **(B)** and pT3 and 4 **(C)**. Comparison of STAM1 protein level in ccRCC patients with low and high tumor grades **(D)** and Kaplan–Meier analysis of OS in patients with grade 1 and 2 **(E)** and grade 3 and 4 **(F)**. Comparison of STAM1 protein level in ccRCC patients with low and high AJCC stages **(G)** and Kaplan–Meier analysis of OS in patients with AJCC stage I and II **(H)** and AJCC stage III and IV **(I)**.

Similarly, we also compared the STAM1 protein level between different ccRCC T stages (pT1 and 2 *vs*. pT3 and 4), tumor grade (grade 1 and 2 *vs*. grade 3 and 4), and AJCC stages (stage I and II *vs*. stage III and IV) in the TM cohort (Figure 4). No significant differences were found in the above-mentioned comparisons due to limited samples in the ccRCC groups. However, the outcomes of OS analyses in ccRCC patients indicated that low STAM1 level was an ideal biomarker in predicting worse survival in early-stage ccRCC patients (pT1 and 2: *P* < 0.0001; grade 1 and 2: *P* < 0.0001; AJCC stage I and II: *P* < 0.0001) (Figure 4).

### Prognostic Value of STAM1 in ccRCC Patients' Survival

Univariate and multivariate Cox regression analyses were conducted to evaluate the association between ccRCC patients' OS and clinical parameters including age, gender, TNM stage, AJCC stage, and STAM1 expression (Table 2). Based on a univariate model, significant correlations were determined between OS and age (hazard ratio, HR = 1.02, 95% CI: 1.02–1.04, *P* = 0.010), pT stage (HR = 2.96, 95% CI: 1.95–4.50, *P* < 0.001), pN stage (HR = 3.03, 95% CI: 1.57–5.87, *P* = 0.001), pM stage (HR = 4.02, 95% CI: 2.60–6.22, *P* < 0.001), AJCC stage (HR = 3.35, 95% CI: 2.16–5.19, *P* < 0.001), grade (HR = 2.54, 95% CI: 1.60–4.02, *P* < 0.001), and STAM1 expression (HR = 0.42, 95% CI: 0.27–0.64, *P* < 0.001) in the TCGA cohort. After adjustment by a multivariate model, age (HR = 1.03, 95% CI: 1.01–1.05, *P* = 0.006), pM stage (HR = 3.00, 95% CI: 1.75–5.13, *P* < 0.001), grade (HR = 1.67, 95% CI: 1.01–2.77, *P* = 0.048), and STAM1 expression (HR = 0.52, 95% CI: 0.330.84, *P* = 0.007) retained a significant association with OS and were regarded as independent prognostic factors. Meanwhile, in the TM cohort, results from a univariate model revealed that a significant relationship existed between OS and age (HR = 1.06, 95% CI: 1.02–1.10, *P* = 0.002), pT stage (HR = 7.81, 95% CI: 3.22–18.97, *P* < 0.001), pN stage (HR = 20.91, 95% CI: 5.47–79.90, *P* < 0.001), AJCC stage (HR = 9.00, 95% CI: 3.81–21.24, *P* < 0.001), grade (HR = 4.98, 95% CI: 2.18–11.39, *P* < 0.001), and STAM1 expression (HR = 0.10, 95% CI: 0.04–0.22, *P* < 0.001). However, only AJCC stage (HR = 23.47, 95% CI: 1.07–515.14, *P* = 0.045), grade (HR = 3.02, 95% CI: 1.08–8.42, *P* = 0.03), and STAM1 expression (HR = 0.12, 95% CI: 0.04–0.32, *P* **<** 0.001) were identified as independent prognostic factors after a multivariate analysis.

**Table 2:** Univariate and multivariate Cox logistic regression analyses of OS in The Cancer Genome Atlas (TCGA) and TM cohorts.

**Covariates**	**Univariate analysis**			**Multivariate analysis**		
	***P*-value**	**HR**	**95% CI**	***P*-value**	**HR**	**95% CI**
**TCGA cohort**						
Age	0.010	1.02	1.01–1.04	0.006	1.03	1.01–1.05
Gender (ref. female)	0.940	1.02	0.67–1.55	–	–	–
pT stage (ref. T1–T2)	<0.001	2.96	1.95–4.50	0.410	1.41	0.62–3.20
pN stage (ref. N0)	0.001	3.03	1.57–5.87	0.340	1.43	0.69–2.98
pM stage (ref. M0)	<0.001	4.02	2.60–6.22	<0.001	3.00	1.75–5.13
Grade (ref. G1–G2)	<0.001	2.54	1.60–4.02	0.048	1.67	1.01–2.77
AJCC stage (ref. I–II)	<0.001	3.35	2.16–5.19	0.690	1.21	0.47–3.09
STAM1 expression (ref. low)	<0.001	0.42	0.27–0.64	0.007	0.52	0.33–0.84
**TM cohort**						
Age	0.002	1.06	1.02–1.10	0.450	1.01	0.98–1.05
Gender (ref. female)	0.150	2.21	0.75–6.46	–	–	–
pT stage (ref. T1–T2)	<0.001	7.81	3.22–18.97	0.110	0.09	0.00–1.70
pN stage (ref. N0)	<0.001	20.91	5.47–79.90	0.540	1.73	0.30–9.84
pM stage (ref. M0)	–	–	–	–	–	–
Grade (ref. G1–G2)	<0.001	4.98	2.18–11.39	0.030	3.02	1.08–8.42
AJCC stage (ref. I–II)	<0.001	9.00	3.81–21.24	0.045	23.47	1.07–515.14
STAM1 expression (ref. low)	<0.001	0.10	0.04–0.22	<0.001	0.12	0.04–0.32

*OS, overall survival; TM, tissue microarray; HR, hazard ratio; CI, confidence interval*.

### STAM1 Inhibited Cell Growth and Invasion in ccRCC Cell Lines

**Figure 5:**
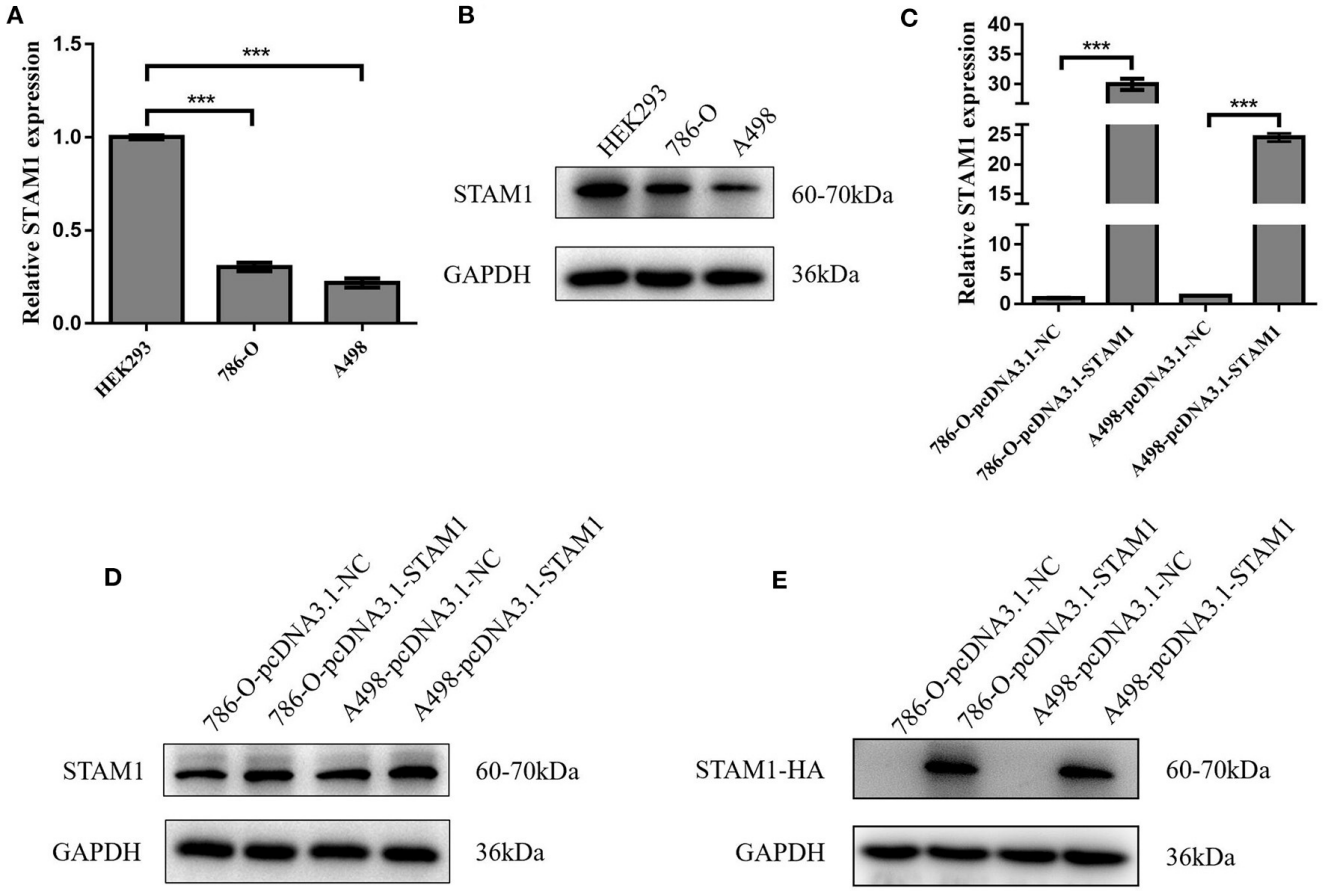
mRNA and protein expression of STAM1 in normal kidney cell and clear cell renal cell carcinoma (ccRCC) cell lines and the verification of STAM1 overexpression in ccRCC cell lines. Comparison of mRNA **(A)** and protein **(B)** expression of STAM1 in HEK293, 786-O, and A498 cells. Verification of STAM1 overexpression in mRNA **(C)** and protein **(D,E)** levels in 786-O and A498 cells. ***p < 0.001.

**Figure 6:**
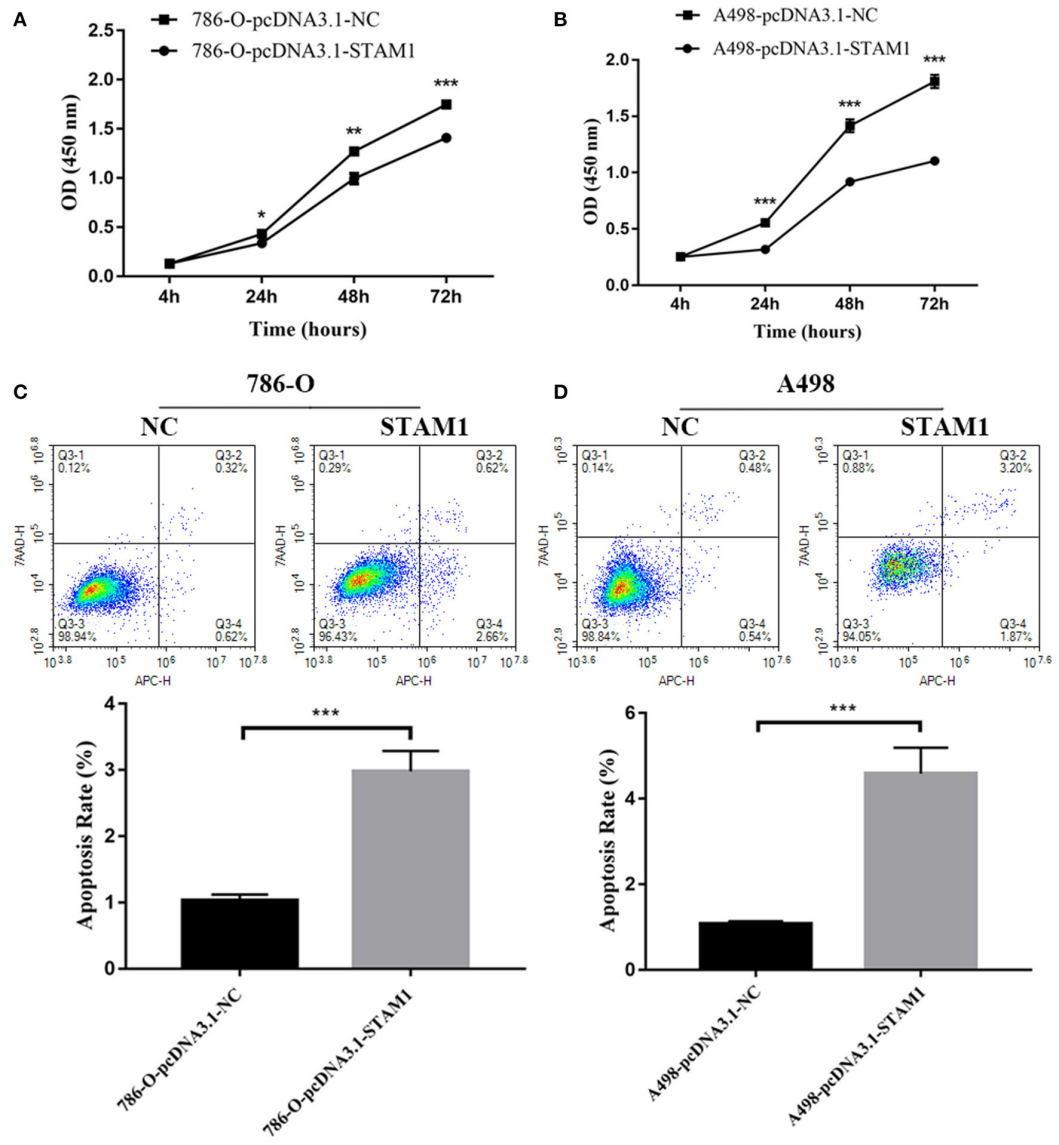
Cell viability and apoptosis assessment after overexpressing STAM1 in 786-O and A498 cells. The cell viability of 786-O **(A)** and A498 **(B)** cells was examined by the cell counting kit-8 assay. The apoptosis rates of 786-O **(C)** and A498 **(D)** cells were evaluated by Annexin V-APC/7-AAD double-staining method and flow cytometry. *p < 0.05, **p < 0.01, and ***p < 0.001.

**Figure 7:**
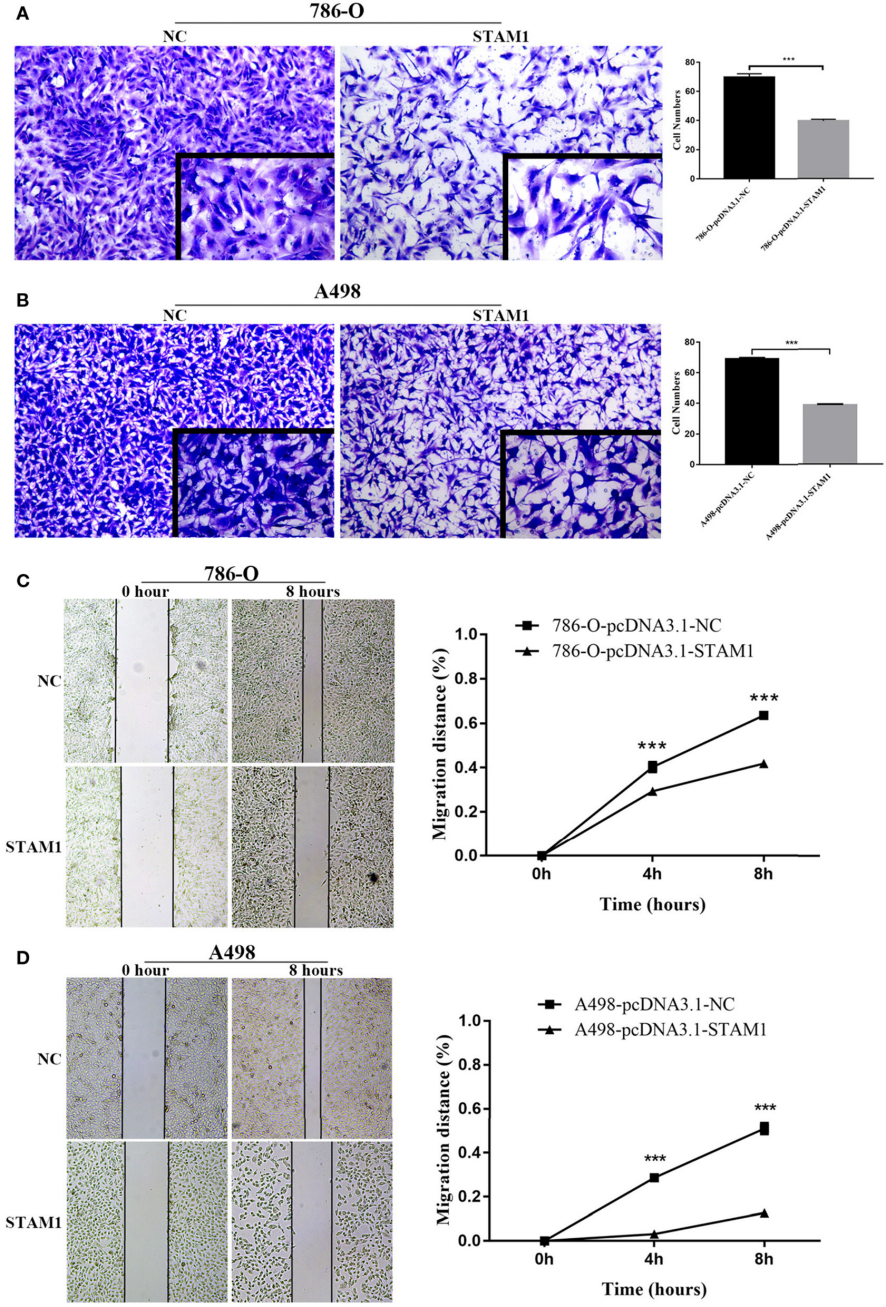
Invasion and migration capability assessment after overexpressing STAM1 in 786-O and A498 cells. The invasion capability of 786-O **(A)** and A498 **(B)** cells was determined using the Matrigel transwell invasion assay. At 24 h later, the cells that had passed though the membrane were calculated and compared to baseline levels. The motility of 786-O **(C)** and A498 **(D)** cells was detected by the wound-healing assay. Migration distances compared to baseline were measured after 4 and 8 h. ***p < 0.001.

We conducted a series of experiments to further explore how STAM1 modulated the progression of ccRCC *in vitro*. Firstly, STAM1 expression was detected in HEK293, 786-O, and A498 cell lines. A significantly lower expression of STAM1 was found in both 786-O and A498 cells compared with HEK293 in mRNA (Figure 5A) and protein level (Figure 5B, Supplementary Figure 1). Then, 786-O and A498 cell lines were transfected with a plasmid overexpressing STAM1 or a control vector, and the successful overexpression of STAM1 is shown in Figures 5C–E and in Supplementary Figure 2. Then, in the cell viability assay, 450 nm OD value decreased in both 786-O- (Figure 6A) and A498-pcDNA3.1-STAM1 (Figure 6B) cell lines compared with controls at each time point (4, 24, 48, and 72 h). Accordingly, the percentage of cell apoptosis was higher in ccRCC cells when STAM1 was overexpressed (Figures 6C,D). In addition, Transwell assay demonstrated that higher STAM1 expression reduced the invasion ability of 786-O and A498 cell lines (Figures 7A,B). Finally, results of the wound-healing assay proved that overexpression of STAM1 prevented the migration of these two ccRCC cell lines (Figures 7C,D).

### Identification and Function of the STAM1 PPI Network

**Figure 8:**
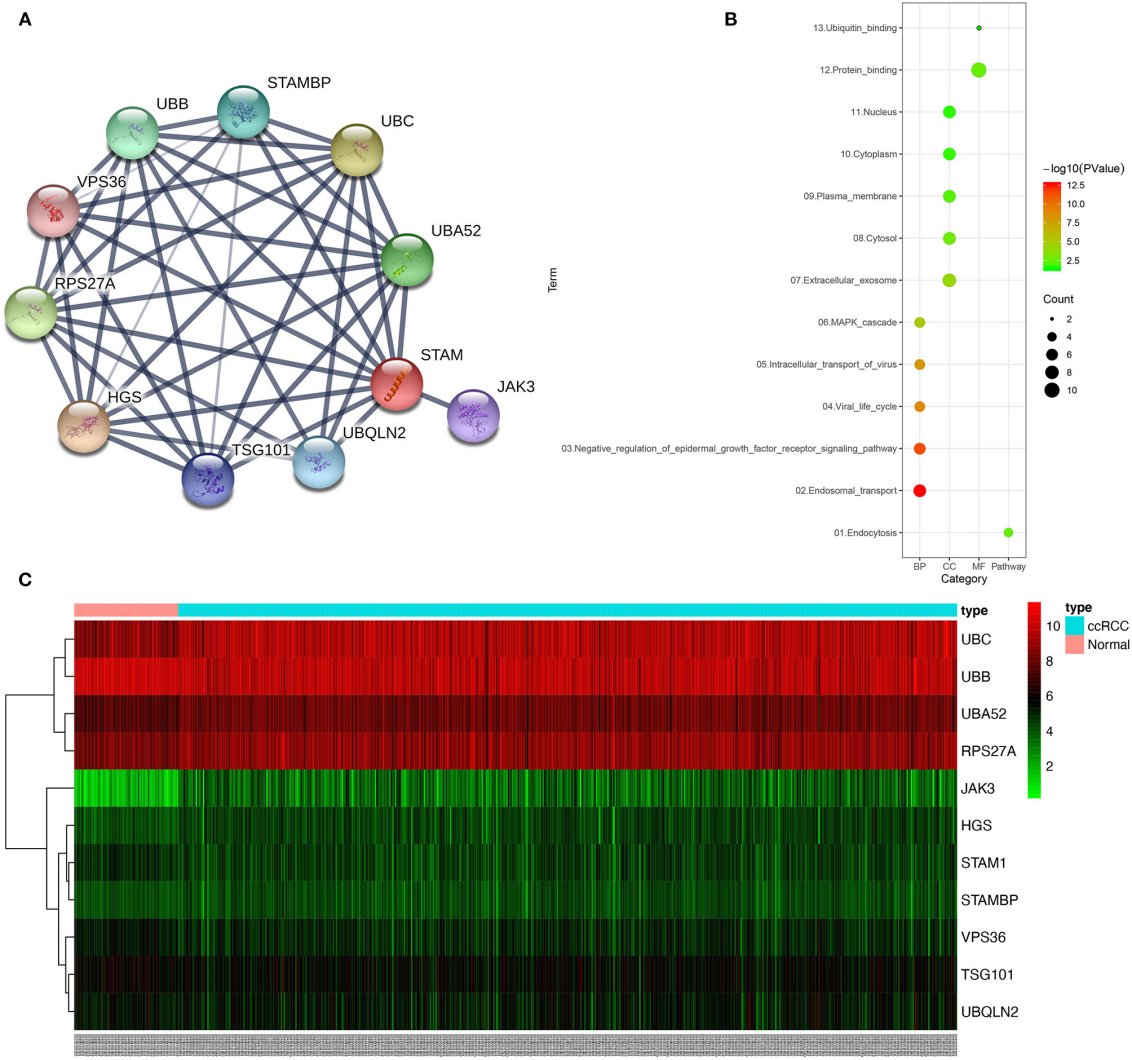
Protein–protein interaction (PPI) network, gene ontology (GO) functional annotation, and Kyoto Encyclopedia of Genes and Genomes (KEGG) pathway enrichment analyses of genes correlated with STAM1. The PPI network identified the top 10 genes correlated with STAM1 **(A)**. GO functional annotation and KEGG pathway enrichment analysis of genes included in the PPI network **(B)**. Heatmap of the top 10 genes correlated with STAM1 in TCGA **(C)**.

The construction of PPI network using STRING online database identified the top 10 genes significantly related to STAM1, namely, HGS, UBC, RPS27A, UBA52, UBB, STAMBP, UBQLN2, TSG101, JAK3, and VPS36 (Figure 8A, Supplementary Table 3). To determine the biological functions of STAM1 as well as its 10 interrelated genes, GO functional annotation and KEGG pathway enrichment analyses were performed as presented in Figure 8B. GO analysis indicated that these 11 genes were significantly involved in five biological processes (“endosomal transport,” “negative regulation of epidermal growth factor receptor signaling pathway,” “viral life cycle,” “intracellular transport of virus,” and “autophagy”), five cellular components (“extracellular exosome,” “cytosol,” “plasma membrane,” “cytoplasm,” and “nucleus”), and two molecular functions (“protein binding” and “ubiquitin binding”). A detailed category of these genes is listed in Supplementary Table 4 based on the above-mentioned biological processes. KEGG analysis revealed that these genes were significantly enriched in the “endocytosis” pathway (Supplementary Figure 3). The expression of STAM1 and its 10 closely related genes from 72 normal kidney samples and 539 ccRCC samples in the TCGA cohort was visualized and plotted as heatmap, as shown in Figure 8C and Supplementary Figure 4.

## Discussion

In this study, we investigated a novel biomarker, STAM1, and its association with ccRCC for the first time. At the beginning, we screened RNA-seq results from TCGA and ICGC databases, detecting a lower expression of STAM1 in ccRCC tissues compared with normal controls. Downregulation of STAM1 was further elaborated to indicate worse prognosis as an independent factor according to our TM cohort analyses. Finally, we confirmed these findings by a series of *in vitro* experiments using two RCC cell lines overexpressing STAM1, demonstrating that STAM1 inhibited RCC cells' viability and functions.

Although most ccRCC patients receive partial or total nephrectomy as a primary treatment, still nearly one-third of them will have tumor recurrence (25). Predicting the prognosis of ccRCC precisely is necessary to help clinicians understand the disease at a relatively earlier stage and then instructing patients to receive further close follow-up and/or adjuvant therapies (26). The application of molecular biomarkers provides new insights for ccRCC's treatment and prognostic prediction, such as proliferation markers (Ki-67 and PTEN) and hypoxia-inducible factor members (vascular endothelial growth factor) (9, 27). Some of them were found to be related to prognosis to some extent. However, none of their pathogenesis on ccRCC was validated thoroughly, especially without combining with other prognostic factors, so no such molecular marker has been used extensively in patients as a recommendation (10). Therefore, we concentrated on a target protein, STAM1, as a new prognostic biomarker in malignant tumors. It was discovered in 1996 and hypothesized to play a role in tumorigenesis because of its function in mediating cell proliferation and signaling transduction (11–13).

The association between STAM1 and any of the tumors has not been investigated yet. We firstly demonstrated that low STAM1 expression was a predictor for worse ccRCC prognosis according to bioinformatic analyses of databases, TM cohort evidence, and experimental research *in vitro*. Of note is the fact that the STAM1 mRNA and protein presented slightly different prognostic implications that low mRNA level predicted worse OS in late-stage ccRCC, while low protein level prognosed worse OS in early-stage disease. Possible explanations may include (1) in the TM cohort, the patients with advanced ccRCC were not enough (11, 36, and 12 patients for pT3 and 4, grade 3 and 4, and stage III and IV ccRCC, respectively), which may lead to false-negative results, and (2) the difference between transcriptional and translational biomarkers may result in such an inconsistency, hinting that both mRNA and protein of STAM1 could serve as useful biomarkers for ccRCC and yet have different prognostic meanings. Hence, future cohorts with more samples and detailed clinicopathological data are required for the validation of our results and hypotheses.

In our *in vitro* experiments, the overexpression of STAM1 in q-PCR did not fully reflect a similar increase in protein level (Figure 5). To explain this result, we added a western blot analysis by using a tagged HA to distinguish endogenous and exogenous STAM1. A higher expression of STAM1-HA was found compared with STAM1 after being overexpressed. This result matched the q-PCR more accurately and demonstrated that fast elimination (for example, by proteosome degradation) of STAM1 possibly existed in ccRCC cells, further indicating that STAM1 expression was lower in ccRCC than in normal cells.

According to cell apoptosis assay, we found the apoptotic rate as 3% after STAM1 was overexpressed, which was three times that of the control group in 786-O cells. Similarly, in A498 cells, the apoptotic rate was five times that of the control group after STAM1 was overexpressed. These results showed that apoptosis of RCC cells increased significantly when STAM1 was highly expressed. However, the total apoptotic rate was only 3–5% in cells, causing a relatively lower impact on cell proliferation.

STAM1 is a member of ESCRT proteins, and ESCRT is considered to control cell growth, cytoskeletal changes, and tumor suppression (15, 28). The ESCRT machinery consists of five complexes: ESCRT-0, ESCRT-I, ESCRT-II, ESCRT-III, and AAA-ATPase complex Vps4 (15, 29). It mainly functions by recruiting one another in a specific sequence to modulate the formation of a multivesicular body and the sorting of ubiquitinylated membrane proteins to lysosomes (30). The dysregulation or mutation of the ESCRT system is linked to hyperproliferation of cells, apoptosis failure, and tumorigenesis (31). For example, ESCRT was examined to attenuate several tumor-related cell signalings, including RTK and Notch (32, 33), and to maintain cytoskeletal organization.

As a component of ESCRT-0, STAM1 has the potential to participate in the formation and progression of tumors. ESCRT-0, also named STAM-Hrs complex, initiates the recognition of ubiquitinated proteins for sorting (34). One of the most important involved pathways is epidermal growth factor receptor (EGFR) signaling. Depletion of Hrs by knocking down its RNA expression pointed out that EGFR accumulated in cells, further activating MAPK/ERK signaling and its downstream receptors (32, 35). A mutant Hrs also resulted in abnormal EGFR degradation, causing similar pathological processes (36). In our GO analysis, negative regulation of the EGFR pathway was closely related to STAMI PPI network, which was in accordance with previous publications. Besides that, JAK had the strongest negative correlation with STAM1 expression in our bioinformatic statistics, and Scoles et al. illustrated that overexpression of Hrs reduced the activation of the JAK/STAT pathway (37). In this way, STAM1 showed promising value in inhibiting tumor growth *via* different cell signalings. Furthermore, other ESCRT members, which were proved to join tumor formation by the above-mentioned pathways, were also highlighted in our PPI network. TSG101, an ESCRT-I protein, played extensive roles in modulating cell proliferation, apoptosis, actin remodeling, and EGFR and JAK/STAT in cancers (30, 38, 39). Another significant ESCRT-II member, VPS 37, was downregulated in advanced prostate cancer and mediated cell functions based on *in vitro* experiments (40). The expression of RPS27, which was highly expressed in ccRCC tissues compared with controls, promoted proliferation and inhibited the apoptosis of leukemia cells (41). These published evidence ensured our future studies on the mechanisms of STAM1 as an initial ESCRT member to predict the prognosis of ccRCC.

Several limitations should be addressed in our study. First, as stated above, the main purpose of this study is to confirm the function of STAM1 to be a novel predictor for ccRCC patients' prognosis. We conducted a series of experiments *in vitro* to investigate the influence of STAM1 overexpression on RCC cells and explored some potential mechanisms between STAM1 and ccRCC through bioinformatics. Detailed mechanisms will be illustrated in our further studies. Second, due to lacking adequate evidence on related mechanisms, the role of STAM1 might be overinterpreted to some extent based on existing bioinformatic statistics. Furthermore, because the clinical data in our study was from retrospective cohorts, the validity of our results was restrained compared with prospective trials.

## Conclusions

STAM1 was expressed lower in ccRCC, and STAM1 was also an important prognostic biomarker for ccRCC patients' survival. Cell experiments demonstrated that STAM1 inhibited RCC cells' growth, migration, and invasion. Further detailed pathogenesis of STAM1 in modulating RCC progression will be investigated based on candidate functional genes from our bioinformatic analyses.

## Data Availability Statement

The datasets presented in this study can be found in online repositories. The names of the repository/repositories and accession number(s) can be found in the article/Supplementary Material.

## Ethics Statement

The studies involving human participants were reviewed and approved by The Ethics Committee of the First Affiliated Hospital of Guangzhou Medical University, China. The patients/participants provided their written informed consent to participate in this study.

## Author Contributions

TD conceived of the study, participated in the data collection and analysis, participated in the cell experiments, and drafted the manuscript. ZH participated in the data collection, analysis, and drafted the manuscript. XD, DG, CC, WW, YL, and GZ edited the manuscript. All authors contributed to the article and approved the submitted version.

## Conflict of Interest

The authors declare that the research was conducted in the absence of any commercial or financial relationships that could be construed as a potential conflict of interest.
