# Corrigendum: STAM Prolongs Clear Cell Renal Cell Carcinoma Patients’ Survival *via* Inhibiting Cell Growth and Invasion

**DOI:** 10.3389/fonc.2021.726290

**Published:** 2021-09-08

**Authors:** Tuo Deng, Zihao He, Xiaolu Duan, Di Gu, Chao Cai, Wenqi Wu, Yongda Liu, Guohua Zeng

**Affiliations:** Department of Urology and Guangdong Key Laboratory of Urology, The First Affiliated Hospital of Guangzhou Medical University, Guangzhou Medical University, Guangzhou, China

**Keywords:** stam, clear cell renal carcinoma, prognosis, cell growth & apoptosis, invasion

In the original article, there was a mistake in the histogram of **Figure 7B** as published. **The columns of “A498-pcDNA3.1-NC” and “A498-pcDNA3.1-STAM1” were listed inversed**. The corrected **Figure 7** appears below.

**Figure 7:**
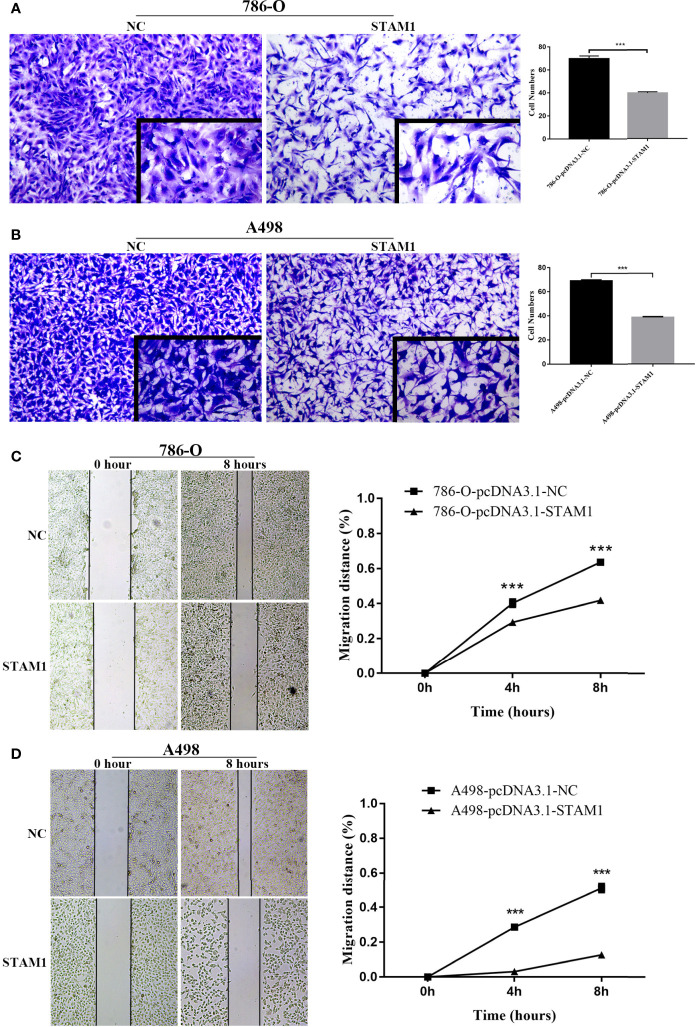
Invasion and migration capability assessment after overexpressing STAM1 in 786-O and A498 cells. The invasion capability of 786-O **(A)** and A498 **(B)** cells was determined using the Matrigel transwell invasion assay. At 24 h later, the cells that had passed though the membrane were calculated and compared to baseline levels. The motility of 786-O **(C)** and A498 **(D)** cells was detected by the wound-healing assay. Migration distances compared to baseline were measured after 4 and 8 h. ***p < 0.001.

The authors apologize for this error and state that this does not change the scientific conclusions of the article in any way. The original article has been updated.

## Publisher’s Note

All claims expressed in this article are solely those of the authors and do not necessarily represent those of their affiliated organizations, or those of the publisher, the editors and the reviewers. Any product that may be evaluated in this article, or claim that may be made by its manufacturer, is not guaranteed or endorsed by the publisher.

