# Supplementary material for: STAM Prolongs Clear Cell Renal Cell Carcinoma Patients' Survival via Inhibiting Cell Growth and Invasion
Source: Front Oncol. 2021 Apr 20;11:611081. doi: 10.3389/fonc.2021.611081 (PMC8093442; doi:10.3389/fonc.2021.611081)
Supplement: Supplementary file 1 [file Data_Sheet_1.PDF]

Supplementary material 1. Supplementary Figures and Tables

Supplementary Figure 1: Original files of western blotting for Figure 5B

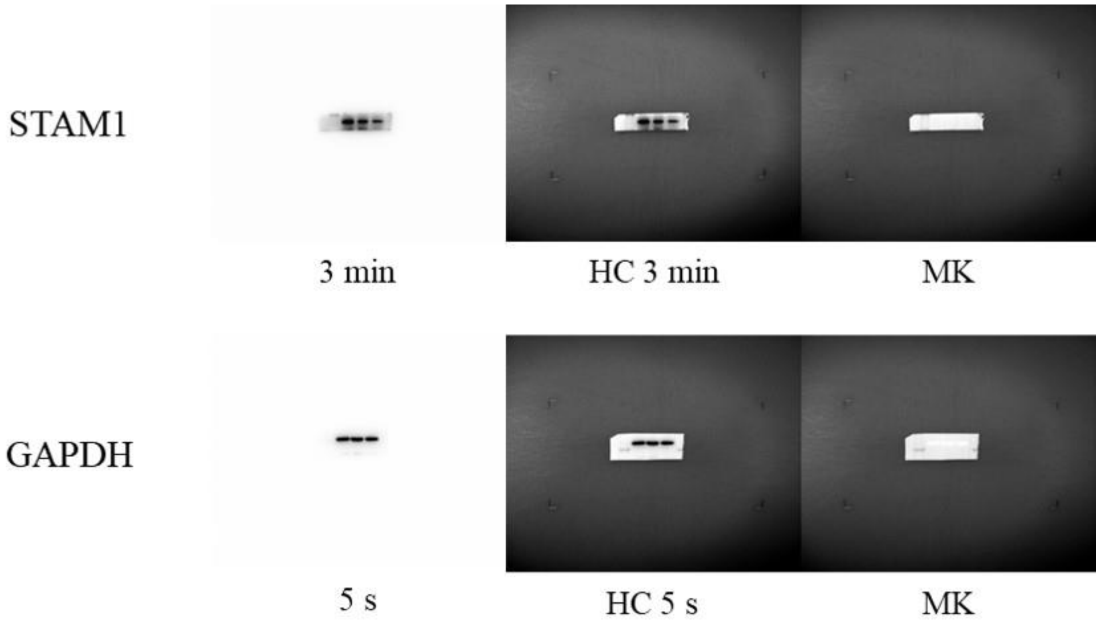

Supplementary Figure 2: Original files of western blotting for Figure 5D and 5E

Figure 5D:

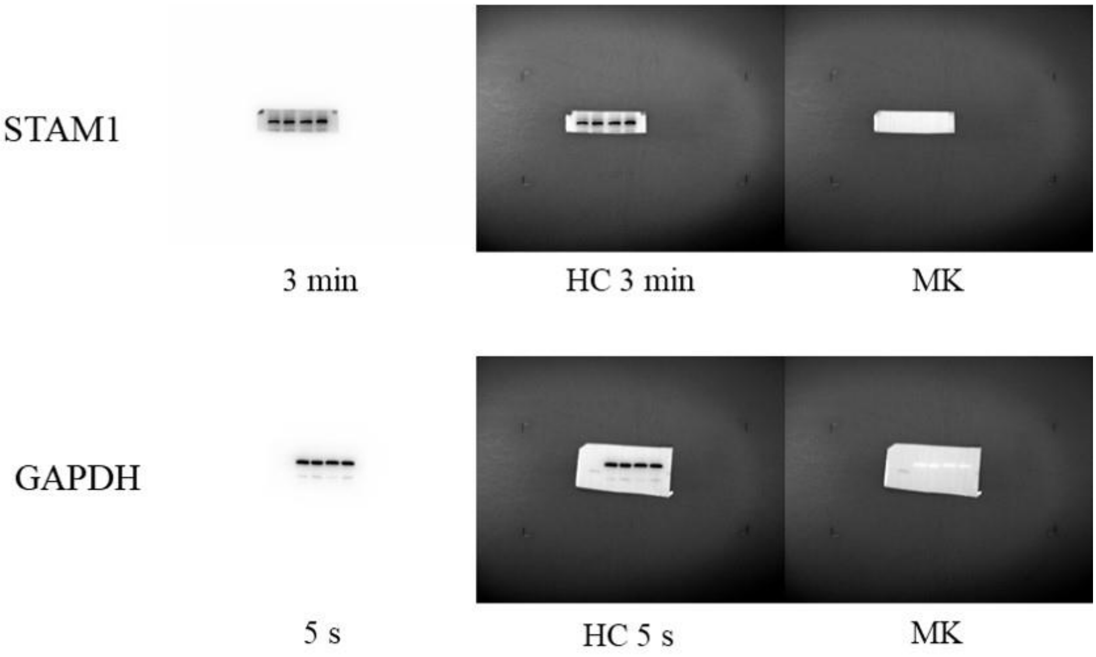

Figure 5E:

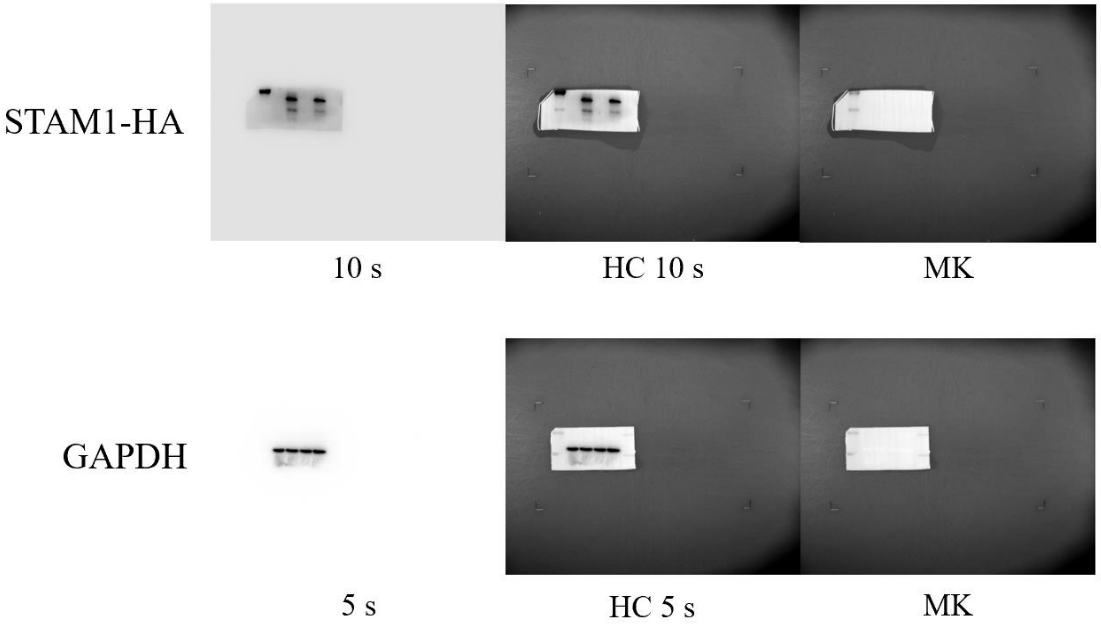

Supplementary Figure 3: The KEGG pathway of endocytosis and endosome transport

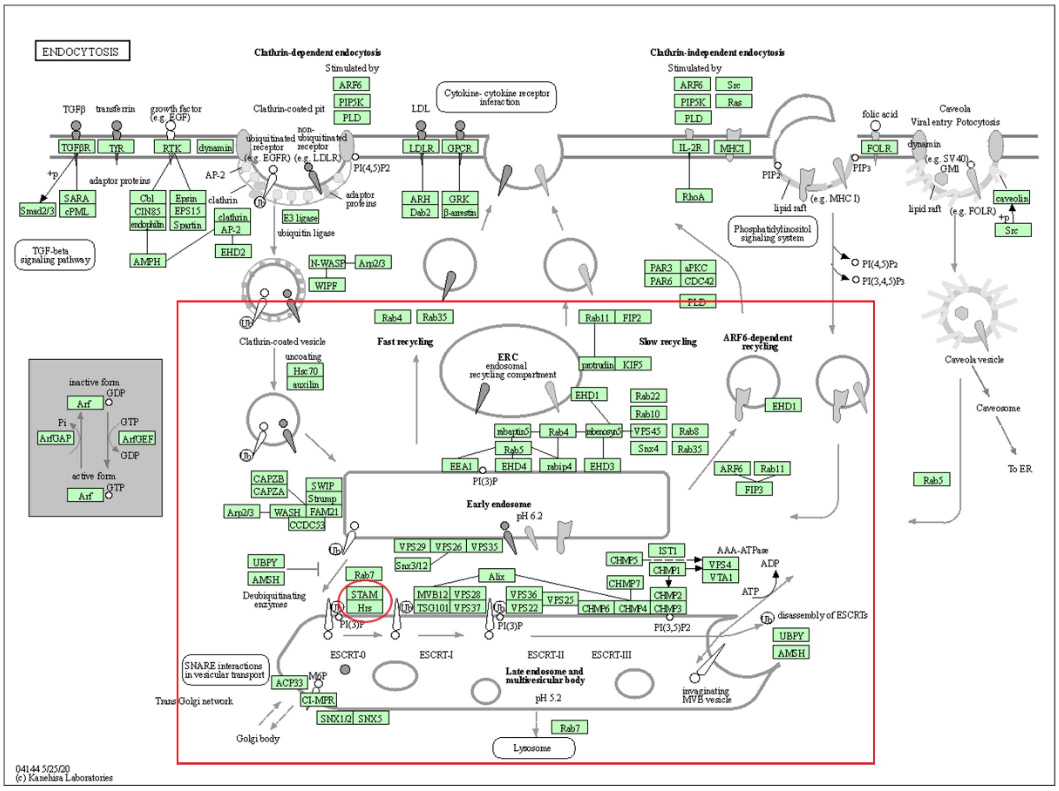

Supplementary Figure 4: Bar plots of expression of the top 10 genes correlated with STAM1 based on TCGA data

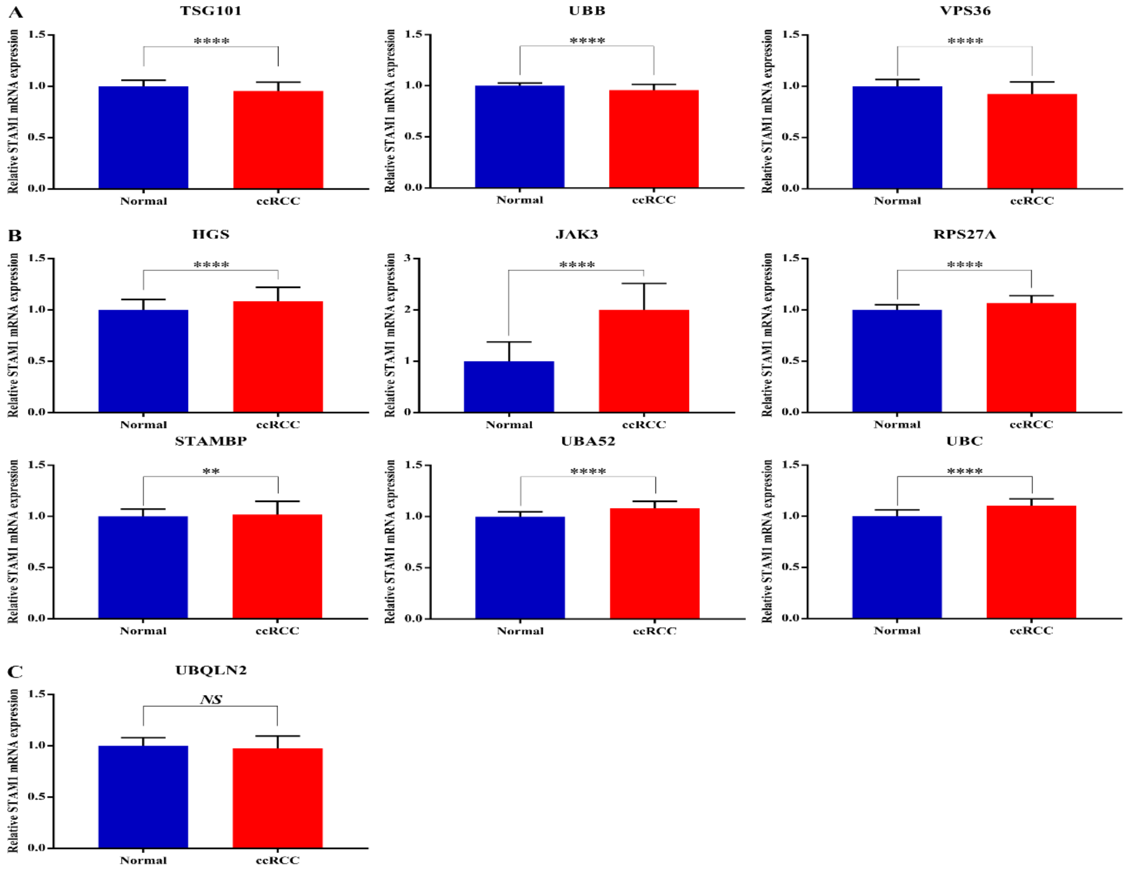
A
TSG101
UBB
VPS36
B
HGS
JAK3
RPS27A
STAMBP
UBA52
UBC
C
UBQLN2
Relative STAM1 mRNA expression
Normal
ccRCC
****
**
NS
0.0
0.5
1.0
1.5
0
1
2
3

Supplementary Table 1. STAM1 expression levels in TCGA and TM ccRCC cohorts

		TCGA	***TM (IHC scores)***
STAM1	Median	4.67	2.52
	Mean	4.54	2.04
	Cutoff	4.10	1.00

In TM cohort, STAM1 expression was semi-quantified by IHC scores, which were integers ranged from 0 to 12.

Supplementary Table 2. ccRCC patients' information at our hospital

	Age	***Gender***	***Laterality***	***Site***	***Size (cm)***	***pT stage***	***pN stage***	***pM stage***	***AJCC Stage***
Patient 1	71	Male	Left	Lower pole	7*5*4	T3	N0	M0	III
Patient 2	45	Male	Right	Mid-upper pole	5.5*4*3.5	T1b	N0	M0	I
Patient 3	60	Male	Right	Lower pole	4.2*3.8*2.7	T1b	N0	M0	I
Patient 4	67	Male	Right	Upper pole	6*5	T1b	N0	M0	I
Patient 5	52	Male	Left	Multiple Metastases	NA	T4	N0	M1	IV
Patient 6	54	Female	Left	Lower pole	4.7*4.3*4.7	T1b	N0	M0	I

ccRCC: clear cell renal cell carcinoma; NA: not available.

Supplementary Table 3. The top 10 hub genes/proteins and their combined scores with STAM1 in PPI network analysis

Gene/Protein	***Combined score***
HGS	0.998
UBC	0.997
RPS27A	0.996
UBA52	0.995
UBB	0.995
STAMBP	0.992
UBQLN2	0.985
TSG101	0.982
JAK3	0.979
VPS36	0.973

Supplementary Table 4. Detailed information of GO functional annotation and KEGG pathway enrichment analyses of genes correlated with STAM1

Category	***Term***	***Gene Count***	**P *value***	***Genes***
Biological process	GO: 0016197~endosomal transport	7	< 0.0001	TSG101, UBC, HGS, UBB, VPS36, UBA52, RPS27A

Biological process	GO: 0042059~negative regulation of epidermal growth factor receptor signaling pathway	6	< 0.0001	TSG101, UBC, HGS, UBB, UBA52, RPS27A
Biological process	GO: 0019058~viral life cycle	5	< 0.0001	TSG101, UBC, UBB, UBA52, RPS27A
Biological process	GO: 0075733~intracellular transport of virus	5	< 0.0001	TSG101, UBC, UBB, UBA52, RPS27A
Biological process	GO: 0000165~MAPK cascade	5	< 0.0001	UBC, UBB, JAK3, UBA52, RPS27A
Cellular component	GO: 0070062~extracellular exosome	8	< 0.0001	STAMBP, TSG101, UBC, HGS, UBB, VPS36, UBA52, RPS27A
Cellular component	GO: 0005829~cytosol	7	0.0018	UBC, HGS, UBB, JAK3, VPS36, UBA52, RPS27A
Cellular component	GO: 0005886~plasma membrane	7	0.0059	STAMBP, TSG101, UBC, UBQLN2, UBB, UBA52, RPS27A
Cellular component	GO: 0005737~cytoplasm	7	0.0201	STAMBP, TSG101, UBC, HGS, UBQLN2, UBB, RPS27A
Cellular component	GO: 0005634~nucleus	7	0.0241	STAMBP, UBC, UBQLN2, UBB, VPS36, UBA52, RPS27A
Molecular function	GO: 0005515~protein binding	10	0.0028	STAMBP, TSG101, UBC, HGS, UBQLN2, UBB, JAK3, VPS36, UBA52, RPS27A
Molecular function	GO: 0043130~ubiquitin binding	2	0.0408	TSG101, VPS36
KEGG pathway	hsa04144: Endocytosis	4	0.003	STAMBP, TSG101, HGS, VPS36
